# Effects of commercial hatchery processing on short- and long-term stress responses in laying hens

**DOI:** 10.1038/s41598-019-38817-y

**Published:** 2019-02-20

**Authors:** Louise Hedlund, Rosemary Whittle, Per Jensen

**Affiliations:** IFM Biology, AVIAN Behavioural Physiology and Genomics Group, Linköping University, 58183 Linköping, Sweden

## Abstract

In commercial egg production, chicks are exposed to a potentially stressful procedure during their first day of life. Here, we investigated how this procedure affects the chickens in a short- as well as long-term perspective by conducting two behaviour tests and measuring corticosterone (CORT) and sex hormone levels at different time points. These results were compared with a group of control chickens from the same hatchery and incubator that did not go through the commercial hatchery routine. Chickens were continuously weighed, egg production data was collected and feather scoring was performed. We found that chicks have a significant increase in CORT during the hatchery process, which implies they are exposed to stress. During first weeks of life, these chicks were more fearful, had a higher CORT reactivity during restraint and weighed more than control chicks. Later in life, hatchery treated chickens had more feather damages and injuries on combs and wattles, a faster onset of egg laying and higher levels of estradiol. We conclude that processing at the commercial hatchery was a stressful event with short- and long-term effects on behaviour and stress reactivity, and potentially also positive effects on production. The results are relevant for a large number of individuals, since the chicken is by far the globally most common farm animal.

## Introduction

In large-scale commercial egg production, laying hen chicks are exposed to a range of potentially stressful procedures during their first day of life. This includes hatching in noisy incubators, manual sex sorting, vaccination and transport to the rearing farms. It affects a large number of individuals, since the chicken is by far the most common farm animal: the world population of egg layers is estimated to between 4 and 5 billion animals^1^. There is little knowledge regarding the extent of stress this may cause in day-old chicks and to what extent it can further affect their behaviour and physiology. The present study is a first attempt at assessing short- and long-term consequences of commercial hatchery routines in laying hen chicks.

The stress response is triggered when facing a perceived threat. As one consequence, the hypothalamic-pituitary-adrenal-axis (HPA-axis) is activated, leading to the release of glucocorticoids, mainly cortisol and corticosterone (CORT), from the adrenal glands^2^. CORT has several short- and long-term impacts on the body, and is commonly used as an indicator of stress^2^. From a behavioural point of view, an animal is defined as ‘stressed’ when it performs behaviours that differ from the normal repertoire^3^. In chickens, examples of this can be altered feeding activity^4^, feather pecking^5^ and aggressive behaviour^6^. Stress can also affect other aspects of behaviour such as ability to cope with novel events, commonly evaluated by novel arena and open field tests^7^. In chickens, tonic immobility (TI) is regularly used for evaluating fear since stressed individuals generally stay longer in this state than non-stressed birds^7^. CORT release during restraint is another well-known measure of stress responsiveness^8^.

Stressful events occurring early in development can affect animals in several ways throughout life^9^. Chicks are sensitive to temperature fluctuations, and early life cold- as well as heat stress has been shown to result in elevated plasma corticosterone concentrations^4^ and depressed weight gain^4,10^ up to at least 35 days of age^11^. Early feed restriction has been shown to affect later body weight in birds such as Japanese quail^12,13^, broiler chickens^14,15^, turkeys^16^, zebra finches^17^ and White Leghorn chickens^18^. However, early stress can also prepare an individual for later challenges and in such way result in a beneficial effect later in life. This is referred to by different terms, such as stress priming, conditioning, stress hardening or hormesis^19,20^ and does not only help the individual to cope better with a particular stressor later in life but can also generalise across stressors^21–23^. For example, in chickens, early thermal stress does not only enhance the ability to cope with temperature fluctuations later in life^4,10,24,25^ but also increases growth and feed efficiency^4,24,25^.

Also the pre-hatch environment can affect the chick in several ways. Chicks exposed to light during incubation perform more feather pecking than chicks brooded in darkness^26^ and incubation temperature has been shown to affect both hatchability and plasma corticosterone levels in the bird^27^. Reduced gas exchange during incubation impairs hatchability^28^, body weight^29^ and cognitive functions such as learning and memory^29^. Exposure of eggs to corticosterone may have negative influences on imprinting^30^, as well as growth in both juveniles and adult chickens^31^, whereas exposure to testosterone can increase growth^32,33^, begging rate^32,33^, social rank^34^ and aggressive and competitive behaviour^34,35^ in young birds, as well as nuptial plumage^35^ and aggressive^35^ behaviour in adult birds. Although this can be highly relevant with respect to commercial hatching effects, the present study focuses on the processing of chicks post-hatch.

From what we know about early stress in chickens as well as other animals, it is somewhat surprising that there is a shortage of research on the effects of commercial hatchery handling of laying hen chicks during their first hours of life. In this study, we compared layer chicks hatched and treated in a commercial hatchery with control chicks which did not go through the commercial hatchery process. Our aim was to investigate how the procedure in a commercial hatchery affects behaviour and stress sensitivity in short- as well as long-term perspective.

## Method

### Ethical note

All experimental protocols were approved by Linköping Council for Ethical Licensing of Animal Experiments, ethical permit no 50–13. Experiments were conducted in accordance with the approved guidelines.

### Animals and experimental treatment

#### General procedure

All chicks were of the *Lohmann LSL* strain from Lohmann Tierzucht, German grandparental stock. Both experimental and control chicks were from the same parent stock and placed at the same time in the same egg rack in the same incubator.

A detailed description of the experiment is given below. In overall summary, to assess effects of the hatchery procedures, we created a control group by collecting eggs from the commercial hatchery at day 19 of incubation, three days before expected hatch. These eggs were hatched in a small incubator at Linköping University, and the resulting chicks were gently placed in rearing pens and used as control animals. On the day of hatching, we collected chicks from the same commercial incubator as the control birds. After standard processing, they were brought to Linköping University and placed in pens in the same room as the control chicks. From this point, control and hatchery processed chicks were treated in the same way until culling at 20 weeks of age. Behavioural and physiological measurements were done at different time periods as outlined below. A total of 83 control chicks and 85 hatchery treated chicks were included in the experiment after 1 week of testing.

#### Hatchery treated chicks (HC)

At day 22 following start of incubation (i.e., when the birds were taken out of the incubators), 130 day-old chicks were obtained from a commercial hatchery (Gimranäs AB, Herrljunga, Sweden). These chicks went through the conventional hatchery process which started 22 days earlier when fertilized eggs arrived at the hatchery and were placed in large cabinet incubators. At day 18, the eggs were moved to hatching trays and placed in hatchers for the last days of incubation. The major part of the eggs hatched at day 21 but the hatchery routinely leaves them in the hatcher until day 22 to maximise hatching rate. After removal from the hatcher, the racks with chicks were tilted on a conveyer belt and the shells removed by hand, which took approximately 3 minutes per rack. The chicks were then moved by another conveyer belt to a sex sorting station where they were manually sexed by wing inspection. The usual hatchery procedure includes discarding of males immediately after sexing, however for this study, males were further processed in the same manner as females since we were interested in chicks of both sexes for this experiment. After the sex sorting, chicks were transported via the conveyer belt system to a vaccination station where they were vaccinated against Marek’s disease by automatic dispensing machines. The vaccination and the sex sorting lasted for approximately 5 minutes each. Once vaccinated, animals were moved to another conveyer belt system with multiple drops including accelerating speed in order to spatially separate chicks for efficient machine counting. Chicks were then automatically counted and dropped into transport boxes. This last phase of the hatchery process lasted for approximately 2 minutes.

Twenty animals were sacrificed in the hatchery for blood samples for corticosterone analyses, 10 immediately after being taken out of the hatchers (stage 1), and an additional 10 after the completion of the hatchery process, i.e. when the birds had been loaded into transport cages (stage 2). The rest of the animals, an equal number of males and females, were transported for 3.5 hours to Linköping University and an additional 10 animals were sacrificed after the transport (stage 3) for blood samples for corticosterone analyses. HC were wing tagged and sham vaccinated at the same time as the control chickens were marked and vaccinated at 8 days of age.

#### Control chickens (CC)

130 eggs were collected at day 19 of incubation from the same commercial hatchery as HC and transported for 3.5 hours to Linköping University in a portable incubator maintaining a temperature of 37 °C. The eggs were placed in a hatching machine set to 37.5 °C and 55% humidity. In total, 113 of the eggs hatched and the chicks were removed from the hatcher at exactly the same time as the HC were taken out of the incubator in the commercial hatchery. A total of 30 animals were sacrificed for blood samples for corticosterone analyses at the exact same time points as the hatchery treated chickens: 10 chicks immediately after removal from hatcher, 10 chicks 15 min later (corresponding to the time point of the end of the hatchery processing), and 10 chicks at the same time as the hatchery chicks arrived from the transport. CC were wing tagged and vaccinated for Marek’s disease at an age of 8 days.

### Housing

Chicks were kept in four identical pens. HC and CC were kept separately but sex mixed in the same groups throughout the whole experiment (pen A CC, females n = 18, CC males n = 25; pen B CC, females n = 23, males n = 17; pen C HC, females n = 14, males n = 28; pen D HC, females n = 21, males n = 22). The animals were initially held in pens measuring 90 × 90 cm, and pen size was doubled after two weeks. At five weeks of age, the chickens were moved to the university animal facility “Wood-Gush” and were placed in pens measuring 1 × 3,5 m for each group. The chickens were kept on saw dust and provided with ad lib food and water. All chickens had access to perches from 1 week of age and to nests from 16 weeks of age.

### Recordings

We collected behavioural, hormonal and production data on the animals to evaluate how the early stress had affected them in short- and long-term perspectives. Table 1 gives a brief overview of the experimental procedure; however, the recordings are more thoroughly described further on.

**Table 1:** Overview of experimental procedure.

Age (days)	Treatment group	
−22	HC + CC	Eggs inserted in incubator at commercial hatchery
−3	HC	Eggs moved from incubator to hatcher at the commercial hatchery
	CC	Eggs collected from the hatchery and placed in a hatcher at the university
0	HC	Chickens taken out of commercial incubator and processed through the commercial hatchery process and transported for 3,5 h. 30 chickens were sacrificed for blood samples
	CC	Chickens taken out of incubator and placed in home pen. 30 chickens were sacrificed for blood samples.
1	HC + CC	Novel arena 1
4	HC + CC	Tonic immobility 1
6	HC + CC	Restraint test 1
35	HC + CC	Chickens moved to another animal facility by 15 minutes car transport
36	HC + CC	Novel arena 2
39	HC + CC	Tonic immobility 2
41	HC + CC	Restraint test 2
105	HC + CC	Blood sampling for sex hormone analysis
133	HC + CC	Blood sampling for sex hormone analysis
105–140	HC + CC	Egg collection
140	HC + CC	Feather scoring and culling
8, 15, 22, 29, 36, 43, 50, 57, 64, 71, 78, 85, 99 and 113	HC + CC	Weighing

#### Restraint test and blood sampling for hormone analysis

Restraint tests were conducted at 6 days of age (HC, n = 21; CC, n = 21) and at 41 days of age (HC, n = 24; CC, n = 22). The chickens were selected randomly from the home pens and a blood sample was taken from the brachial vein within 3 minutes of capture in order to establish a baseline level of CORT^36^. The chickens were then suspended in a net bag for 3 minutes after which a second blood sample was taken to measure CORT increase. 3 minutes of restraint has earlier been shown to elicit a measurable CORT response in chickens^36–38^. Blood samples were also taken at days 105 (females HC, n = 10; females CC, n = 10; males HC, n = 10; males CC, n = 10) and 133 (sample 2; females HC, n = 10; females CC, n = 10; males HC, n = 10; males CC, n = 10) for sex hormone (estradiol and testosterone) analysis.

All blood samples were collected using a microcuvette heparin coated tube which held 200 µl of blood. The blood samples were stored on ice or in a refrigerator until ready for centrifuging in the lab. The plasma was separated and frozen in storage at −40 °C until the time of analysis using a corresponding ELISA test.

#### Novel arena

Novel arena testing (NA) was conducted at an age of 1 day (NA 1; HC, n = 28; CC, n = 28) and repeated at an age of 36 days (NA 2; HC, n = 24; CC, n = 24). The arena (NA 1, 40 × 60 × 25 cm; NA 2, 200 × 200 × 200 cm) contained a fully enclosed start box (NA 1, 15 × 15 × 16 cm; NA 2, 30 × 50 × 30 cm), saw dust, food and water, hay and a novel object (NA 1, a blue pot; NA 2, a glove). All birds were selected randomly from their home pen at the time of testing, hence, birds tested in NA 1 were not necessary the same birds tested in NA 2. In groups of four, individually marked (using a felt-tip pen), chickens were placed in each arena within the start box with a sliding door. In total, 7 groups from each treatment were tested at NA 1 and 6 groups from each treatment at NA 2. The observation started when the door in the start box was opened and continued for 30 minutes. The tests were video recorded, and all videos were analysed with Observer 13 software. We recorded the latency to enter the arena for each bird and the total duration of locomotion for each bird (two or more steps of walking or running).

#### Tonic immobility

Tonic immobility (TI) tests were conducted at 4 (TI 1, HC, n = 26; CC, n = 26) and 39 (TI 2, HC, n = 26; CC, n = 26) days of age. All chickens were selected randomly from their home pen, hence, birds tested in TI 1 were not necessary the same birds tested in TI 2. The birds were carried one at a time into the test room. A bird was placed on its back in a cradle and a light pressure was applied to the body for 10 s. Chickens that righted within 5 s were regarded not to have entered TI and the process was repeated for up to another two times. All tests were performed by the same person. Time of first vocalisation, first head movement and rightening was recorded as well as frequency of vocalisations.

#### Weight and egg production

All chickens were weekly weighed from 1–12 weeks of age and then every second week up to 16 weeks. From when the first egg was laid, eggs were collected daily at the same time of day from each pen. Egg weight and number of eggs per pen each day was recorded. Since it was not possible to have individual recordings of egg laying, we calculated the average numbers of eggs per hen and day for each seven-day period after onset of laying (period 1; period 2; period 3). When comparing treatment effects, we used each day of recording as the statistical replicate.

#### Feather scoring

At 140 days of age, all individuals were scored for feather damage at head, back, belly, wing and tail, and for damage to combs and wattles. Females (HC n = 33; CC n = 38) and males (HC n = 48; CC n = 38) were analysed separately. For the feather scoring, the following scale was used: 0: No damage; 1: A few feathers ruffled with little damages; 2: Many (>5) feathers ruffled with little damages, or missing feathers; 3: Feathers heavily ruffled and major damage to more than one feather, or loss of feathers. For comb and wattle, the following scale was used: 0: No damage; 1: Some (1–2) small bruises; 2: Many (3 or more) bruises of small to severe character; 3: Heavily damaged with many severe bruises and wounds. The same person, who was blind for treatment, performed all feather and comb-wattle scoring.

#### Hormone Analysis

The blood samples from the 1^st^ day as well as from the restraint tests were analysed for corticosterone using ELISA kits from ENZO Life Sciences. The samples were analysed according to the product manual: http://static.enzolifescience.com/fileadmin/files/manual/ADI-900-097_insert.pdf.

Testosterone and Estradiol were analysed using corresponding ELISA kits from MyBioSource. The samples were analysed according to the product manuals: https://www.mybiosource.com/images/tds/protocol_manuals/000000-799999/MBS284852.pdf (estradiol) and https://www.mybiosource.com/images/tds/protocol_manuals/800000-9999999/MBS9424390.pdf (testosterone).

#### Statistical analysis

A Kruskal-Wallis test was used for the tail feather scoring data. Assessment of feather condition at 20 weeks of age showed no variance between or within treatments in the scored values for head, back, belly or wing, therefore we decided to not include these in the analysis. Values for comb and wattle were pooled together in the analysis. The rest of the data was analysed with a generalized linear model (GLM) with treatment and sex as well as their interactions as predictors, except for egg production and gonadal hormones where sex was removed from the model. We used the scale response “linear” and the link function “identity”. The Omnibus test was significant in all cases (p < 0.05). Significance was determined by the Wald χ^2^ – statistics and estimated marginal means were calculated. The differences were considered significant when p < 0.05. For the Novel Arena tests, group means were used as independent replicates. All the statistical testing was done in SPSS.

## Results

### Corticosterone

#### Blood sampling in hatchery

**Figure 1:**
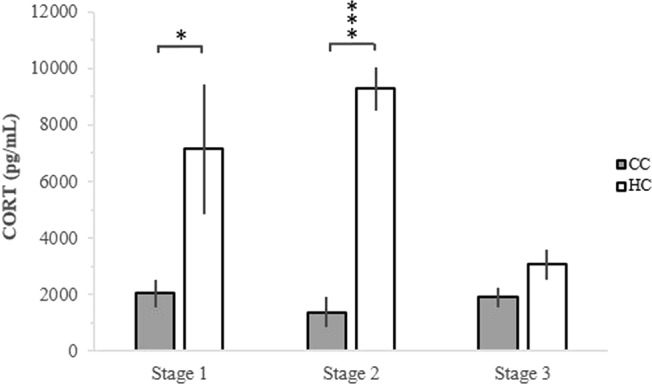
Corticosterone response of hatchery treated chickens (HC) at stage 1 after incubation, stage 2 after the hatchery process and stage 3 after 3.5 h of transport, and control chickens (CC) at corresponding time points. *p < 0.05, ***p < 0.001.

The analysis of blood samples taken during the hatchery and transport process showed that CORT was significantly higher in hatchery treated chickens (HC) compared to control chickens (CC) after stage 1 (χ^2^ = 5.29; df = 1, 18; 10; p = 0.021; Fig. 1). Also after stage 2, there was a significant difference in CORT level between HC and CC (χ^2^ = 75.95; df = 1, 18, 17; p < 0.001; Fig. 1). There was a trend in the same direction for the stage 3 sample, however this was not significant (χ^2^ = 3.69; df = 1, 18; p = 0.055; Fig. 1).

#### Restraint test

**Figure 2:**
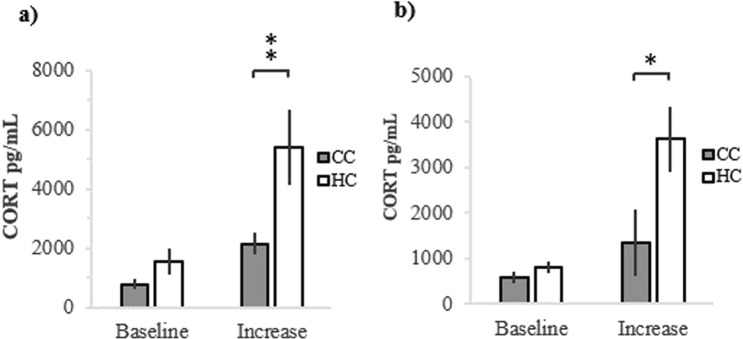
Corticosterone level in hatchery treated chickens (HC) and control chickens (CC) before and after three minutes of restraint at (**a**) 1^st^, and (**b**) 2^nd^ restraint test. **p < 0.01.

The restraint test showed that there was no significant difference between baseline corticosterone levels in HC and CC, either in the 1^st^ (χ^2^ = 2.99, df = 1, 38, p = 0.084; Fig. 2a) or in the 2^nd^ (χ^2^ = 1.39, df = 1, 42, p = 0.238; Fig. 2b) restraint test. However, there was a significant difference between HC and CC in CORT increase during three minutes of stress in restraint, at the 1^st^ (χ^2^ = 6.73, df = 1, 38, p = 0.009; Fig. 2a) as well as the 2^nd^ (χ^2^ = 4.94, df = 1, 42, p = 0.026; Fig. 2b) restraint test.

### Behaviour tests

#### Novel arena

**Figure 3:**
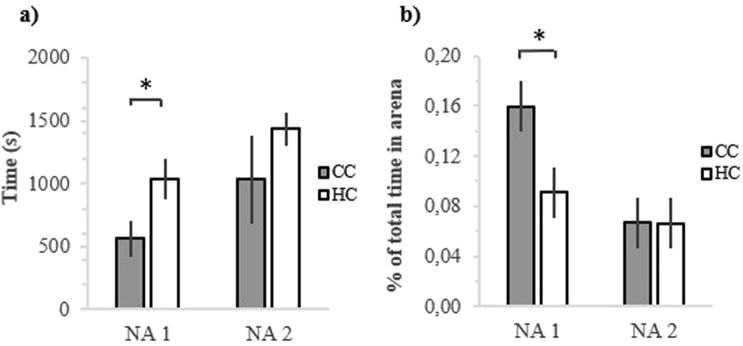
Behaviours of hatchery treated chickens (HC) and control chickens (CC) in novel arena. (**a**) Latency to emerge from start box and enter the arena, in seconds; (**b**) Locomotion behaviour in novel arena, % of total time spend outside the start box in the arena. *p < 0.05.

Novel arena testing (NA-test) showed that there was a statistically significant difference between HC and CC in the latency to emerge from the start box and enter the arena at 1^st^ (χ^2^ = 5.43, df = 1, 12, p = 0.020; Fig. 3a) but not at the 2^nd^ NA-test (χ^2^ = 1.39, df = 1, 10, p = 0.238; Fig. 3a). CC performed significantly more locomotion behaviour in the novel arena than HC at the 1^st^ (χ^2^ = 5.84, df = 1, 12, p = 0.016; Fig. 3b) but not at the 2^nd^ NA-test (χ^2^ = 0.00, df = 1, 10, p = 0.992; Fig. 3b).

#### Tonic immobility

Neither TI 1 nor TI 2 showed a significance difference between HC and CC in latency to rightening (TI 1; HC; 286.1 ± 32.1 s; CC 272.3 ± 31.2 s; χ^2^ = 0.02, df = 1, 48, p = 0.885; TI 2; HC 164.9 ± 23.9 s; CC 243.9 ± 41.8 s; χ^2^ = 2.37, df = 1, 48, p = 0.123), latency to first head movement (TI 1; HC 219.4 ± 30.1 s; CC 236.0 ± 28.1 s; χ^2^ = 0.28, df = 1, 48, p = 0.597; TI 2; HC 236.0 ± 28.1 s; χ^2^ = 1.22, df = 1, 48, p = 0.270), latency to first peep (TI 1; HC 114.1 ± 23.22; CC 161.6 ± 28.4 s; χ^2^ = 1.98, df = 1, 48, p = 0.159; TI 2; HC 40.1 ± 7.6 s; CC 44.7 ± 6.1 s; χ^2^ = 0.17, df = 1, 48, p = 0.677) or number of peeps per second (TI 1; HC 0.3 ± 0.0 peeps/s; CC 0.3 ± 0.0 peeps/s; χ^2^ = 0.86, df = 1, 48, p = 0.355; TI 2; HC 0.5 ± 0.2 peeps/s; CC 0.3 ± 0.1 peeps/s; χ^2^ = 1.50, df = 1, 48, p = 0.221). However, in TI 2, there was a significant sex effect, where females peeped more than males (M 0.2 ± 0.1; F 0.3 ± 0.2; χ^2^ = 4.45 df = 1, 48; p = 0.035) and a significant interaction between sex and treatment (χ^2^ = 4.46, df = 1, 48, p = 0.035) where HC males performed significantly more peeps than HC females (M 0.7 ± 0.1; F 0.1 ± 0.2; χ^2^ = 4.45 df = 1, 48, p = 0.035).

### Weight

HC birds weighed more than CC already at day 8, and the difference between treatments was then maintained throughout the whole experimental period (Table 2). As shown by the absence of interaction effects, both sexes were affected the same way by the hatchery treatment.

**Table 2:** Mean weight in hatchery treated chickens (HC) and control chickens (CC), males (M) and females (F) respectively, in grams from 8–113 days of age.

Days of age	HC		CC		*p*-value, treatment	*p*-value, treatment*sex
	M	F	M	F		
8	81.7 ± 0.8	79.9 ± 1.0	72.2 ± 0.9	70.0 ± 0.9	<0.001	0.800
15	140.2 ± 1.6	136.4 ± 1.9	129.8 ± 1.7	122.8 ± 1.7	<0.001	0.361
22	224.0 ± 2.7	212.0 ± 3.3	212.6 ± 3.0	195.1 ± 3.0	<0.001	0.364
29	328.4 ± 4.05	300.6 ± 4.8	319.3 ± 4.3	284.1 ± 4.4	0.003	0.397
36	436.3 ± 5.1	393.6 ± 6.0	433.1 ± 5.5	375.6 ± 5.6	0.056	0.184
43	532.9 ± 6.4	481.1 ± 7.6	529.3 ± 7.0	461.8 ± 7.1	0.102	0.262
50	634.2 ± 7.6	561.5 ± 9.1	630.0 ± 8.3	545.8 ± 8.4	0.236	0.493
57	684.1 ± 9.8	610.1 ± 11.8	647.1 ± 10.7	562.7 ± 10.9	<0.001	0.634
64	822.9 ± 11.3	704.6 ± 13.4	770.1 ± 12.3	669.8 ± 12.3	<0.001	0.465
71	949.0 ± 12.0	795.7 ± 14.2	909.2 ± 13.3	773.1 ± 13.1	0.018	0.513
78	1072.5 ± 13.7	879.4 ± 16.2	1046.0 ± 15.1	872.7 ± 14.9	0.269	0.510
85	1217.3 ± 16.2	980.4 ± 19.2	1123.1 ± 17.9	936.1 ± 17.7	<0.001	0.161
99	1431.9 ± 15.3	1102.1 ± 18.1	1368.7 ± 17.1	1064.6 ± 16.7	0.003	0.446
113	1646.6 ± 17.2	1245.2 ± 20.4	1596.8 ± 19.1	1234.6 ± 18.8	0.110	0.299

### Gonodal hormones and egg production

**Figure 4:**
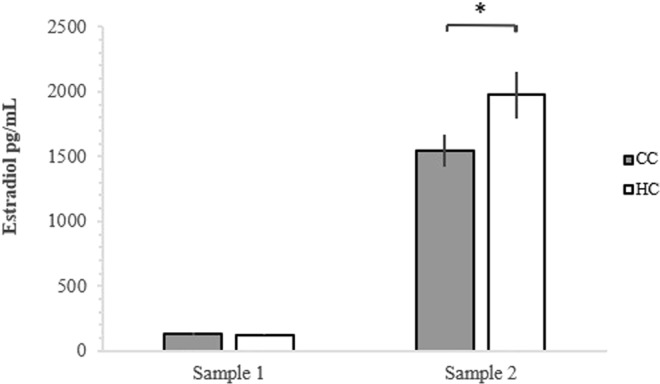
Estradiol levels in hatchery treated chickens (HC) and control chickens (CC) in sample 1 before sexual maturity, and sample 2, after onset of sexual maturity.

The analysis of testosterone levels in males showed no significant difference between HC and CC, neither at the time of first sample before sexual maturity (HC 2987.3 ± 434.52 pg/mL; CC; 2099.38 ± 271.37 pg/mL; χ^2^ = 3.17, df = 1, 18, p = 0.075) nor at the time of the second sample after sexual maturity (HC 6332.28 ± 1428.74 pg/mL; CC 7231.85 ± 1309.28 pg/mL; χ^2^ = 0.23, df = 1, 18, p = 0.634). When comparing estradiol levels in the females between HC and CC, there was no significant difference at the time of first sample before sexual maturity (χ^2^ = 0.35, df = 1, 18, p = 0.553; Fig. 4), however; there was a significant difference in estradiol level between HC and CC at the time of the sampling after onset of sexual maturity, where HC had a higher level of estradiol than CC (χ^2^ = 4.06, df = 1, 18, p = 0.044; Fig. 4).

**Figure 5:**
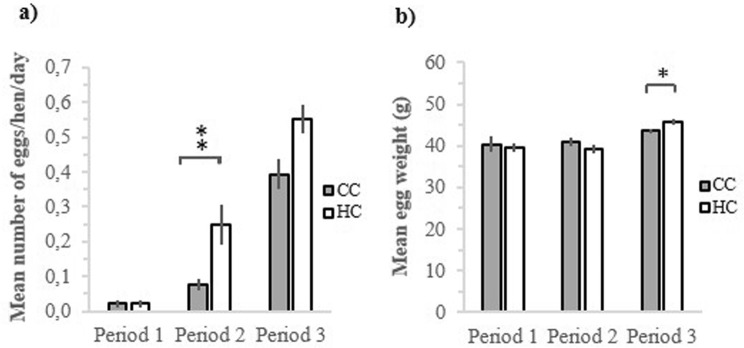
Eggs laid of hatchery treated chickens (HC) and control chickens (CC) during 1^st^ period (day 1–7), 2^nd^ period (day 8–14) and 3^rd^ egg laying period (day 15–22), where day 1 is the day when the first egg was laid. (**a**) Number of eggs laid per hen and day during 1^st^, 2^nd^ and 3^rd^ period. (**b**) Mean weight in eggs laid during 1^st^, 2^nd^ and 3^rd^ period. *p < 0.05, **p < 0.01.

For the egg numbers, we divided the data into three time periods; period 1 (day 1–7), period 2 (day 8–14) and period 3 (day 15–22), where day 1 was the day when the first egg was laid. There was no difference between treatments in age at on-set of egg laying, therefore this parameter was not included in the analysis (First egg laid: HC 119 days old; CC 121 days old). There was no difference between HC and CC in number of eggs laid during 1^st^ (χ^2^ = 0.00, df = 1, 26, p = 0.977; Fig. 5a) or 3^rd^ period (χ^2^ = 3.78, df = 1, 26, p = 0.052, Fig. 5a), however; there was a significant difference between HC and CC in eggs laid during 2^nd^ period (χ^2^ = 9.27, df = 1, 26, p = 0.002; Fig. 5a). Furthermore, there was no significant difference between HC and CC in egg weight during the 1^st^ (χ^2^ = 0.16, df = 1,7, p = 0.694, Fig. 5b) or 2^nd^ period (χ^2^ = 1.06, df = 1, 22, p = 0.303; Fig. 5b), but there was a difference during the 3^rd^ period (χ^2^ = 6.26, df = 1, 30, p = 0.012; Fig. 5b).

### Feather scoring

**Figure 6:**
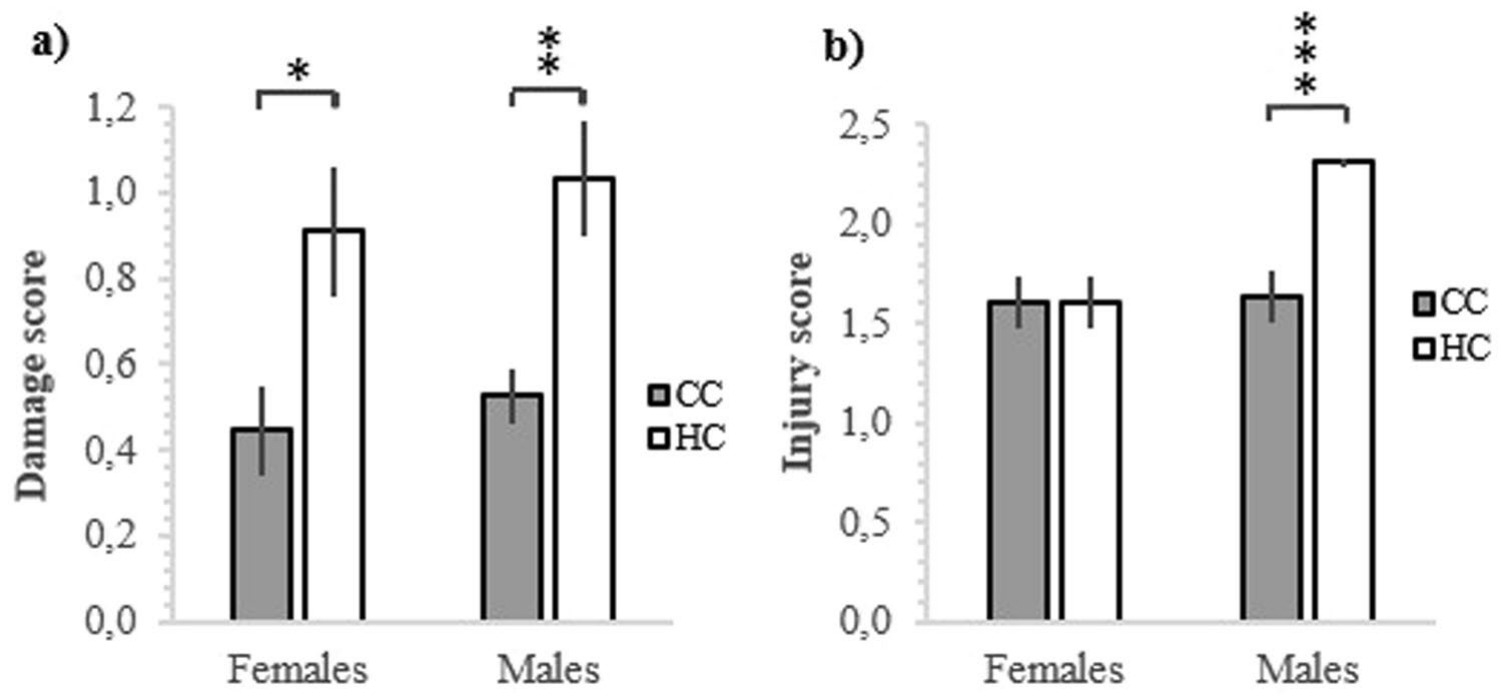
Scores for injuries and damages on hatchery treated chickens (HC) and control chickens (CC) at 20 weeks of age on (**a**) tail feathers, and (**b**) wattle and comb. Higher scores signify more damage.

There was a significant difference between HC and CC in tail feather damages for both males (p = 0.003) and females (p = 0.020, Fig. 6a), where HC had higher damage scores. The difference between HC and CC in scores for wattle and comb was non-significant in females (p = 0.975), however, in males, HC had significantly more damages than CC (p = 0.001, Fig. 6b).

## Discussion

Our results show that commercial hatchery processing affects chickens in several aspects in a short- as well as long-term perspective. Corticosterone (CORT) levels were elevated during and after the hatchery processing, and the CORT reactivity was increased for several weeks in the commercial hatchery chickens (HC). Furthermore, HC showed more fearful behaviour as observed in a Novel Arena test. However, HC weighed more than control chickens (CC) and HC females also reached sexual maturity sooner than CC. This indicates that although the commercial hatchery process caused a significant and prolonged stress response, it may also have had long-term positive effects for the birds, perhaps through stress priming. In the following paragraphs, the results are discussed in more detail.

HC had enhanced levels of circulatory CORT already after incubation and an explanation for this can be that commercial incubators contain large fans to circulate the air which as a result cause high levels of noise. The effects of exposure to noise during development is to our knowledge not very well investigated in chickens, however, other environmental pre-hatch factors have been shown to have major impacts on the birds post-hatch^26,27,29^. We also found enhanced levels of CORT after the hatchery process including separation from shells, conveying, sex sorting, vaccination and packaging, which shows that the hatchery process is indeed causing an activation of the HPA-axis. There are several aspects of the hatchery process that can play a role in this. Earlier studies have shown that human handling^39^, noise^40^, light^41^, temperature^4,10^ and pain^42^ can all be sources of stress in young chicks. Further research is needed to disentangle the roles of each of these potential stressors.

We found no significant increase in CORT level after transport. This can either be a result of a depletion or exhaustion of the HPA-response^2^, or it may indicate that the transport situation is in fact not perceived as particularly challenging to the birds. This is in contrast to what is known from broiler research where blood corticosterone level has been reported to substantially increase during transport^43–45^. However, the broiler research is conducted on full-grown birds and therefore, the comparison should be treated with care. It is also possible that broiler chickens and layers differ in their stress reaction to transport since these breeds have been intensely selected for different purposes during the last century. In the restraint test, there was no significant difference in baseline CORT levels between HC and CC. However, HC showed higher level of circulatory CORT after restraint than CC at both the test instances. This shows that HC had a higher HPA-axis reactivity to a stressful event than CC. Wang^46^ explored the effect of CORT administration on gene expression relating to HPA-axis activity in broiler chickens and found that less stressed individuals had increased numbers of glucocorticoid receptors in the hypothalamus which indicates a more efficient feedback loop. It is possible that exposure to commercial hatchery stress alters the expression of HPA related genes, for example, glucocorticoid receptors, causing changes in HPA-axis reactivity.

Hatchery processing caused an acute effect on fear related behaviour lasting for at least one week. HC challenged in a novel arena took longer time to enter the arena and were less active than CC. This would imply that HC were more fearful^7,47^, since latency to enter a novel environment as well as reduced activity are considered validated fear responses for chickens^48^. However, by 36 days of age, these differences between the groups had disappeared, which indicates that the fear levels had normalized at this age. We could not find any significant differences between the two groups of chickens in behaviours measured in the tonic immobility (TI) test. TI-responses are mainly related to the immediate experiences of a chicken^7^, so therefore the most severe effects of the hatchery processing may have waned at the time when we performed the test. In future studies, it might be better to perform the TI test after a brief challenge, since this could better reflect hatchery effects on stress responsivity.

In the present study, there was a significant difference in number of wounds and amount of feather damage between HC and CC where HC had more feather damage (females) and wounds on the comb and wattles (males). The feather damage in the females indicates an increased incidence of feather pecking among the HC, whilst the comb and wattle damage indicate increased aggression. Early-life history, laying, brooding and rearing conditions have been shown to have major effects on feather pecking^49^. It is also shown that treating eggs with CORT influences the behaviour of the chick post-hatch^50,51^ and since we found increased CORT levels after incubation, it is possible that the incubation in the commercial hatchery might have contributed to the significant difference in feather pecking later in life.

There was a significant weight difference between HC and CC, where HC weighed more throughout the whole experimental period. In contrast, earlier studies have shown that early life stress can cause decreased weight gain^8,10^, but early life stress in chickens can also increase feed efficiency^52,53^ and thereby body weight^24,25^. Furthermore, there was a difference in egg production regarding both egg number and egg size between HC and CC where HC laid somewhat more and larger eggs. We could also see a difference in estradiol level between HC and CC females where HC females had a significantly higher level of estradiol after sexual maturity than CC. It is well known that egg size is correlated to weight of the hen which can explain our results^54,55^ but earlier studies have shown that stress negatively impacts egg production^56,57^. The results regarding egg production need to be treated with care, since we used deliberate pseudoreplication by basing our analysis on repeated sampling days as replicates. Nevertheless, they suggest that the hatchery stress might have had measurable effects on the reproductive system of the hens almost 20 weeks later. However, the results need to be replicated with proper individual sampling methods.

## Conclusion

In this study, we wanted to evaluate how the handling of day-old layer chicks during the first day of life in the commercial hatchery affects them in a short- as well as long-term perspective. We found that chickens experience stress during the commercial hatchery processing, which may have several effects later in life. Hatchery treated chickens were more fearful to novelty, had a higher CORT reactivity and weighed significantly more than control chickens. Further, hatchery stressed females had higher levels of estradiol after sexual maturity and possibly laid more eggs. They also had more feather damage and injuries on comb and wattle suggesting there was more feather pecking and aggression in this group than in the control group. We conclude that the process in the commercial hatchery is a stressful experience for chickens and that it has short- as well as long-term effects on behaviour, hormonal levels and potentially also production. More knowledge about how commercial hatchery processes affect chickens is needed to improve hatchery routines.

## Data Availability

The datasets generated during and/or analysed during the current study are available from the corresponding author on reasonable request.
